# Simalikalactone E (SkE), a new weapon in the armamentarium of drugs targeting cancers that exhibit constitutive activation of the ERK pathway

**DOI:** 10.18632/oncotarget.791

**Published:** 2012-12-30

**Authors:** Guillaume Robert, Valérie Jullian, Arnaud Jacquel, Clémence Ginet, Maeva Dufies, Stephanie Torino, Anaïs Pottier, Frederic Peyrade, Sophie Tartare-Deckert, Geneviève Bourdy, Eric Deharo, Patrick Auberger

**Affiliations:** ^1^ INSERM/U1065, C3M, Nice, France; ^2^ Equipe 2: Morts Cellulaires, Différenciation, inflammation et Cancer; ^3^ Equipe labellisée par la Ligue Nationale Contre le Cancer 2011-2013; ^4^ Université de Nice, France; ^5^ Equipe 6: Toxines microbiennes dans la relation hôte-pathogènes; ^6^ Université de Toulouse; Université Paul Sabatier; Pharma-Dev UMR 152; Faculté de Pharmacie; 35 Chemin des maraîchers, F-31062, Toulouse cedex 9, France; ^7^ Institut de Recherche pour le Développement (IRD); Pharma-Dev UMR 152; Faculté de Pharmacie, 35 Chemin des maraîchers, F-31062, Toulouse cedex 9, France; ^8^ Equipe 11, Microenvironnement, Signalisation et Cancer; ^9^ Centre Antoine Lacassagne, Nice, France

**Keywords:** Simalikalactone E, B-Raf inhibitor, CML, Melanoma, Hairy Cell Leukemia, ERK pathway

## Abstract

Simalikalactone E (SkE) is a quassinoid extracted from a widely used Amazonian antimalarial remedy. Although SkE has previously been shown to have cytostatic and/or cytotoxic activities in some tumor cell lines, its mechanism of action has not yet been characterized. We show here that SkE in the high nanomolar range inhibited the growth of various leukemic and solid tumor cell lines. Importantly, SkE was highly efficient at inhibiting chronic myelogenous leukemia (CML) cells that exhibit constitutive activation of the MAPK pathway and, accordingly, it impaired the phosphorylation of ERK1/2. SkE also abrogated MEK1/2 and B-Raf phosphorylation but had no effect on Ras activity. Moreover, SkE was particularly effective against melanoma cell lines carrying the B-Raf-V600E mutation. Importantly, SkE resensitized the PLX-4032-resistant 451Lu melanoma cell line (451Lu-R) and was more efficient than U0126, a MEK inhibitor, and PLX-4032 (PLX) at inducing the apoptosis of two Hairy Cell Leukemia (HCL) patient samples carrying the B-Raf-V600E mutation. Finally, SkE was as efficient as imatinib at inhibiting tumor formation in a xenograft model of CML cells in athymic mice. In conclusion, we show that SkE, a very potent inhibitor of B-Raf-V600E, is highly effective against cancer cell lines that exhibit constitutive activation of the ERK1/2 pathway.

## INTRODUCTION

Simalikalactone E (SkE) is a new quassinoid extracted from a widely used Amazonian antimalarial remedy derived from *Quassia amara* L. (Simaroubaceae) leaves. In the mid-nanomolar concentration range, this new molecule inhibits the growth of Plasmodium falciparum cultured in vitro by 50%, independent of the strain sensitivity to chloroquine. SkE can also decrease gametocytemia when present at a 50% inhibitory concentration seven fold lower than that of primaquine, a leading compound for treating malaria. SkE is less toxic than simalikalactone D (SkD), another antimalarial related quassinoid from *Quassia amara*, and its cytotoxicity towards mammalian cells is dependent on the cell line; it displays a good selectivity index when tested on non-tumorigenic cells. In vivo, SkE inhibits murine malarial growth of *Plasmodium vinckei petteri* by 50% at doses of 1 and 0.5 mg/kg body weight/day when administered by the oral and intraperitoneal route, respectively [1]. Furthermore, unpublished data from our laboratories have established that SkE may have potent antileukemic activity on several hematological malignancies.

The Ras/Raf/MEK/ERK pathway is frequently altered in cancer cells, and mutations in this pathway are recurrent in several hematopoietic and non-hematopoietic malignancies [2, 3]. It is also worth mentioning that mutation of an upstream protein in the MAP kinase pathway excludes the possibility of mutation of another protein in the pathway [4, 5]. For instance, N-Ras, one of the upstream regulators of the pathway, is mutated in 20% of melanoma, whereas K-Ras is mutated in 80% of pancreatic carcinoma. B-Raf, an effector of Ras and the upstream kinase in the ERK cascade, is frequently mutated in melanoma (50-70%) [6], Langerhans cell histiocytosis (57%) [7], thyroid carcinoma (40%) [8] and colorectal cancer (8%) [9]. The frequency of B-Raf mutation is generally very low in leukemia; however, it was recently reported that B-Raf is mutated in most cases of HCL [10-12]. Finally, mutations in MEK1 are also detected at a low frequency in melanoma [13]. In all cases, the mutated protein seems to be endowed with constitutive activity. Inhibitors of B-Raf such as PLX have been introduced recently with success as new anti-melanoma agents that can induce complete remission in patients [14]. Unfortunately, resistance to PLX has been found to occur rapidly after the onset of treatment, mainly through reactivation of the MAP kinase pathway [15]. Therefore, it is essential to develop new therapeutic strategies aimed at inhibiting the MAPK pathway in these resistant patients.

Importantly, HCL is another disease characterized by the B-Raf mutation [10]. HCL is a rare leukemia affecting B cells. This hematopoietic malignancy is associated with the B-Raf V600E mutation in most of patients. This hallmark of the disease has provided the rationale for the use of vemurafenib (PLX-4032) in two patients suffering from HCL who had no other therapeutic options [16]; Peyrade et al. 2012 (in press). In both cases, a two-month treatment with the drug led to elimination of the leukemic clone as well as restoration of normal erythrocyte, platelet and leukocyte counts, which were accompanied by a considerable improvement in the patient status.

In the present study, we describe the activity and mechanism of action of SkE, a new natural compound extracted from Quassia Amara that exhibits both potent anti-leukemic and anti-melanoma effects in vitro and in vivo because of its ability to interfere with the ERK cascade. Therefore, SkE should be tested as a new therapeutic option in cancers that exhibit constitutive activation of the ERK pathway.

## RESULTS

### SkE exerts potent antileukemic activity *in vitro*

**Figure 1:**
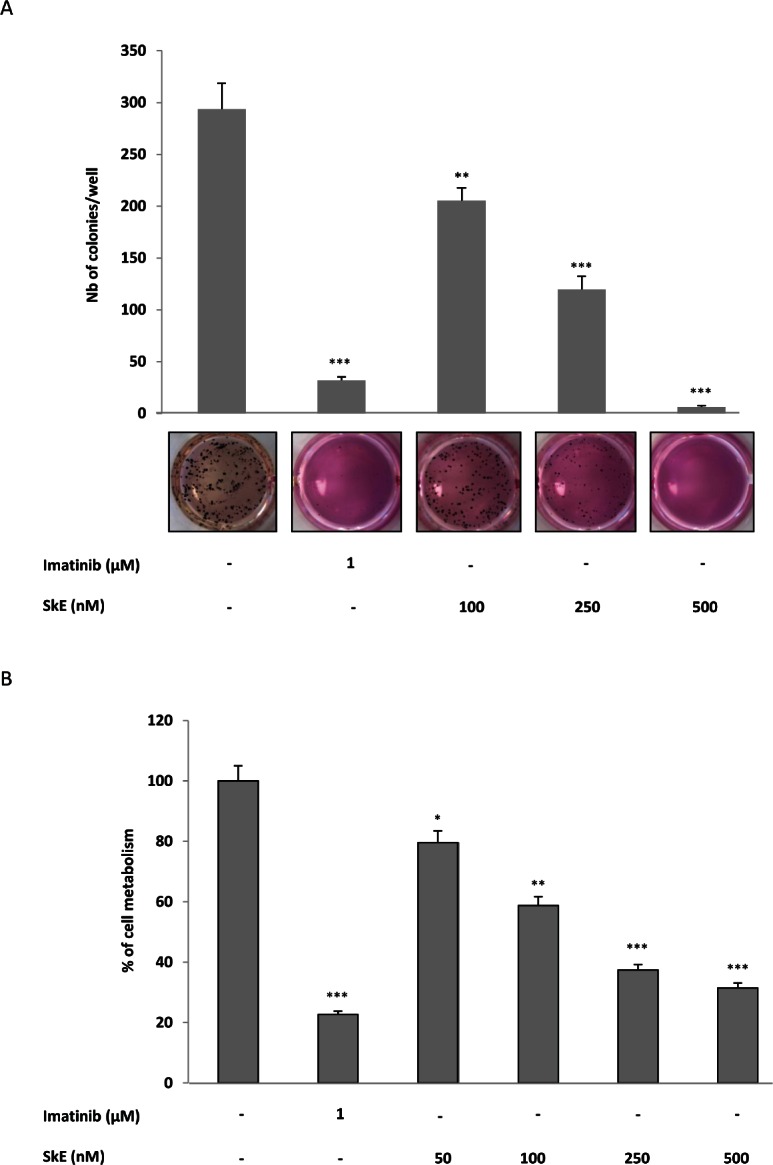
SkE treatment induces cell death of CML cell lines and primary CML CD34+ cells (A) SkE in the 100-500 nM range was added to K562 CML cell lines growing in semi-solid methyl cellulose medium (0.5'10^3^ cells/ml). Imatinib (1 μM) was used as an internal control. Colonies were detected after 10 days of culture by adding 1 mg/ml of MTT reagent and were scored by Image J quantification software. Results are expressed as the number of colony forming cells per well after drug treatment. Results are given as the mean ± SD of 3 different determinations made in triplicate. Error bars = 95% confidence intervals. (B) Primary CML CD34+ cells were incubated for 48 h at 37°C with increasing concentrations of SkE. The cell metabolism was measured by the XTT assay, as described in the Materials and Methods section. Results are given as the mean ± SD of 3 different determinations made in triplicate. Error bars = 95% confidence intervals.

We have reported previously that SkE is both cytostatic and cytotoxic for some tumor cell lines [1]. The present study was conducted to address the mechanism of action of SkE in different cancer cell lines. We first used the well-characterized human K562 cell line to determine whether SkE affects the proliferation of leukemic cells. To this end, we performed colony formation assays in soft-agar using increasing doses of SkE or a maximal dose of imatinib, a tyrosine kinase inhibitor that targets BCR-ABL, the fusion oncoprotein responsible for this disease. As expected, imatinib (1μM) inhibited the clonogenic potential of K562 cells in soft-agar by more than 90% (Figure 1A). Importantly, SkE was a highly potent inhibitor of K562 cell colony formation in identical conditions, with a maximal effect at 500 nM (Figure 1A). At this dose, SkE was even more potent than imatinib, the leading therapy for CML. The IC50 value for the SkE effect was found to be 250 nM. SkE was also a very potent inhibitor of CD34+ cell growth for cells isolated from two CML patients at diagnosis (Figure 1B). Finally, SkE also exerted potent antileukemic effects on several imatinib-resistant CML cell lines (not shown).

### SkE inhibits the MAP kinase pathway

**Figure 2:**
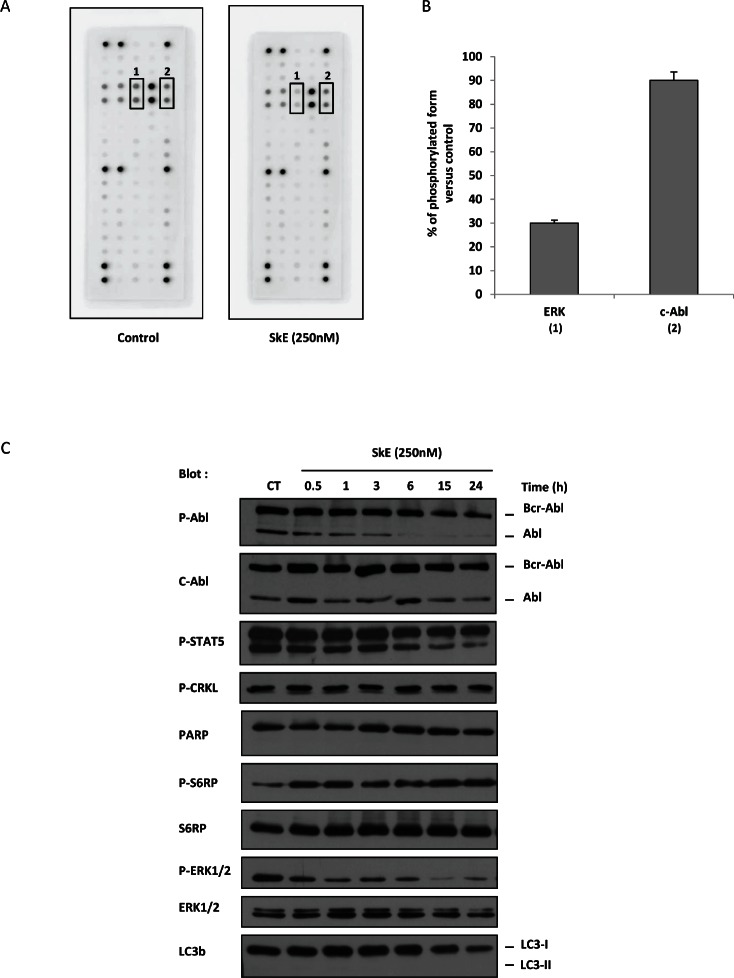
SkE treatment impairs ERK1/2 phosphorylation (A) K562 cells were treated with 250 nM of SkE for 2 h; then, cells were lysed, and cell lysates were loaded on a Pathscan multikinase® membrane. (B) Histograms represent the relative intensity quantification of the most regulated dot with Image J software. Results are expressed as the percentage of kinase phosphorylation in SkE-treated cells versus control cells. (C) K562 cells were incubated at 37°C with 250 nM SkE for the indicated times. Whole-cell lysates were prepared, and the expression of Phospho-C-Abl, C-Abl, Phospho-STAT5, Phospho-CRKL, PARP, Phospho-S6RP, S6RP, Phospho-ERK1/2, ERK1/2 and LC3b was visualized on a Western blot.

In an attempt to identify the potential targets of SkE, we used the PathScan® RTK signaling antibody array kit from Cell Signaling, which allows the simultaneous quantification of the activity of approximately 50 kinases. Among these kinases, two were significantly affected by SkE. Indeed, SkE inhibited the activity of ERK by 70% and c-Abl by 15% (Figure 2 A and B). To confirm the effect of SkE on BCR-ABL activity, we next incubated K562 cells for 2 h with 250 nM of SkE and analyzed the phosphorylation status of both BCR-ABL and known BCR-ABL substrates. In accordance with the results obtained with the RTK signaling array kit, we confirmed the inhibition of c-Abl by SkE as judged by the decreased phosphorylation of c-Abl as soon as 3 hrs after the addition of SkE to the culture medium. We also noted a decrease in the phosphorylation status of STAT5 (Figure 2C). Moreover, dephosphorylation of ERK1/2 was clearly detected as soon as 30 min after the addition of SkE and was maximal at 15 h. Collectively, our results confirm that SkE is a very potent inhibitor of the ERK pathway in K562 cells. Furthermore, it appears that c-Abl dephosphorylation did not precede ERK dephosphorylation but rather followed ERK inhibition. Figure 2C also shows that SkE failed to affect autophagy in K562 CML cells, as assessed by the absence of delipidation of LC3-b in cells treated with this drug.

### SkE is a B-Raf inhibitor

**Figure 3:**
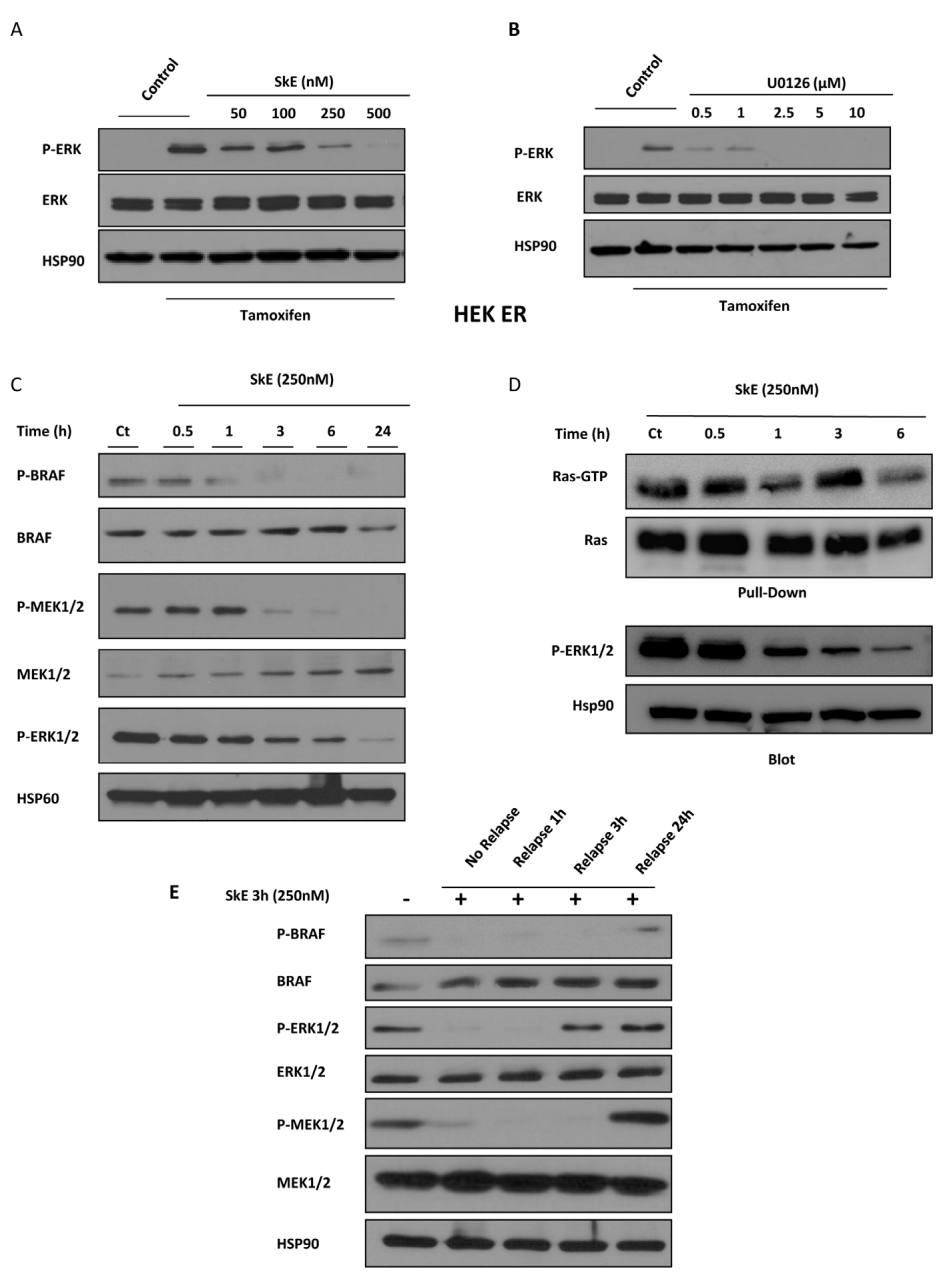
SkE can inhibit the RAF/MEK/ERK signaling pathway *HEK Raf-ER cells* were pre-treated with increasing doses of SkE (A) or U0126 (B) for 1 h. Cells were then treated with tamoxifen (1 μM) for one additional hour. Protein samples were separated by electrophoresis, and the expression of Phospho-ERK and ERK was visualized on a Western blot. (C) K562 leukemic cells were treated for different times with 250 nM SkE. The status of phosphorylation of BRAF, MEK and ERK was visualized by Western blot. (D) K562 cells were incubated at 37°C with 250 nM SkE for the indicated times. Ras activity was determined after GST-pull-down. Ras-GTP levels were determined using GST-c-Raf RBD to pull down active GTP-bound Ras from cell extracts by glutathione beads. The beads were washed 4 times and subjected to SDS/PAGE (12% polyacrylamide). Ras and Phospho-ERK1/2 proteins were detected by Western blot analysis. (E) K562 cells were treated with 250 nM SkE for 3 h. Cells were then washed and placed in fresh medium for 1 h, 3 h or 24 h. The BRAF, MEK and ERK1/2 protein levels and their phosphorylation status were analyzed by Western blot. HSP60 and HSP90 were used as the loading controls.

We next used the ^Δ^Raf-1:ER (HEK-ER) cells, which express an inducible form of the kinase Raf-1, to assess the effects of SkE in comparison with U0126, a well-known inhibitor of MEK1, in the Ras/Raf/MEK/ERK pathway. Tamoxifen induced the activation of the ERK pathway, as assessed by the increased phosphorylation of ERK1/2 (Figure 3A). Importantly, SkE (500 nM) was as efficient as U0126 (2.5 μM) at abolishing tamoxifen-induced ERK1/2 activation (Figure 3B). To precisely identify the target of SkE, we analyzed the entire ERK pathway. SkE efficiently inhibited the phosphorylation status of both MEK1/2 and B-Raf (Figure 3C). However, SkE failed to affect the activity of Ras in a GST-RAS pull-down assay (Figure 3D). Collectively, our data clearly demonstrate that SkE acts as an inhibitor of B-Raf. Finally, the effect of SkE on the ERK cascade was rapidly reversible upon withdrawal of the drug (Figure 3E).

### SkE inhibits the growth of PLX resistant-cell lines *in vitro*

**Figure 4:**
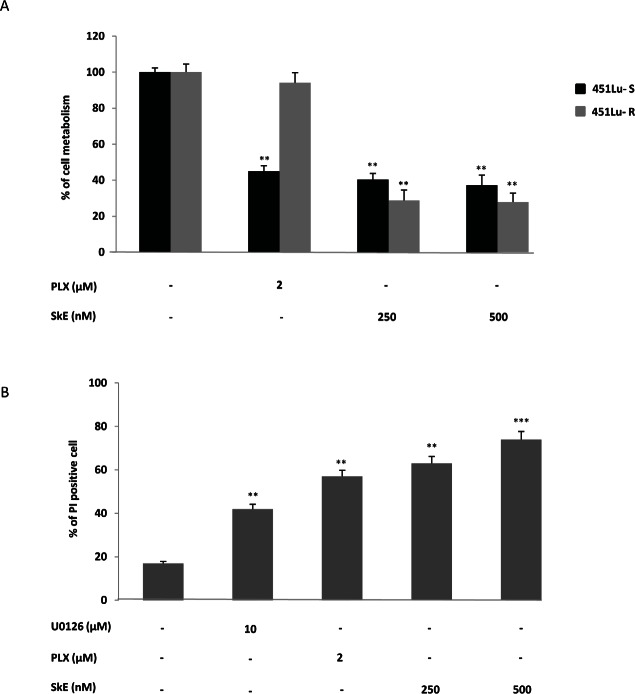
SkE induces cell death of tumors exhibiting the BRAF V600E mutation (A) PLX-sensitive and PLX-resistant 451Lu melanoma cell lines were treated with either 2 μM PLX or increasing doses of SkE. Forty-eight hours later, cell metabolism was measured by the XTT assay as described in the Materials and Methods section. (B) Primary blood cells from patients suffering from hairy cell leukemia were treated with 2 μM PLX, 10 μM U0126 or increasing doses of SkE. Twenty-four hours later, cells were stained using propidium iodide, and cell death was analyzed with a cytometer.

PLX, also known as vemurafenib, has been shown to be highly effective in both B-Raf V600E melanoma cell lines and in patients with metastatic melanoma. However, in patients, the rapid reactivation of the ERK cascade is responsible for relapses. We investigated whether SkE was capable of resensitizing PLX-resistant cell lines.To this end, we used dabrafenib (GSK2118436) sensitive and resistant melanoma cell lines which also exhibits cross resistance to vemurafenib (PLX-4720). This PLX-sensitive 451 melanoma cell line and its PLX-resistant counterpart were incubated for 24 h with PLX (1 μM) or two concentrations of SkE and the cell viability was assessed using the XTT assay. As expected, the 451Lu-R melanoma cell lines [22] were fully resistant to PLX, whereas both the 451Lu-R cell lines were highly sensitive to the effect of SkE (Figure 4A). Importantly, PLX-resistant cells appeared to be even more sensitive to SkE.

We next analyzed the efficiency of U0126, PLX and SkE on blood cells from two HCL patients carrying the B-Raf V600E mutation. SkE, at a concentration of 500 nM, induced cell death in more than 70% of the blood cells, as assessed by propidium iodide staining (Figure 4B), whereas PLX and U0126 were less efficient, triggering 55% and 44% cell death, respectively. As a whole, these findings show that SkE also exhibited high activity against the B-Raf V600E mutation.

### SkE inhibits the growth of CML cells in athymic mice

**Figure 5:**
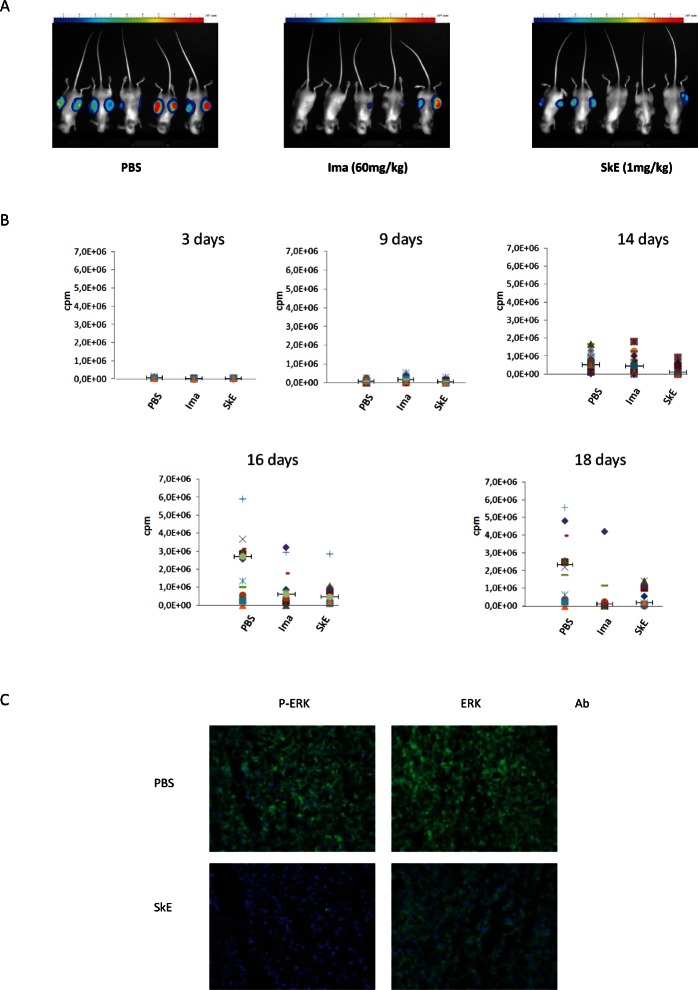
Chronic treatment with SkE is as effective as imatinib in inhibiting the growth of tumors derived from CML cells in athymic mice A total of 5'10^6^ K562 leukemic cells were implanted in both flanks in athymic mice. After tumor establishment, when the tumors reached 100 mm3, animals received a daily intraperitoneal injection of vehicle, imatinib (60 mg/kg body weight) or SkE (1 mg/kg body weight). (A) This picture shows the size of tumors at day 16 post injection with vehicle (left panel), Imatinib (middle panel) and SkE (right panel). (B) This panel shows the evolution of tumor growth, expressed in cpm at different times post-injection of the same treatments. (C) Tumors were removed, frozen and cut to perform immune staining. Slides containing a representative section of each tumor were incubated with either an anti-phospho-ERK1/2 or anti-ERK1/2 antibody. Slides were finally mounted and analyzed under a fluorescence microscope.

To address the efficacy of SkE in vivo, we investigated the ability of the drug to inhibit the growth of the K562 CML cell line implanted in athymic mice. To this end, K562 cells carrying the luciferase gene were injected in the flanks of athymic mice. Mice were randomized and separated into three groups. When tumors reached 100 mm^3^ in size (after 5 days), each subgroup of mice was treated daily with an intraperitoneal injection of vehicle, 60 mg/kg imatinib or 1 mg/kg of SkE. At day 18, imatinib and SkE had induced tumor regression to a similar extent (Figure 5A). The tumor size was evaluated by photon imaging at days 3, 9, 14, 16 and 18 following the injection of 30 mg/kg of luciferin (Figure 5B). The inhibitory effect of SkE on K562 cell growth in vivo was detected as early as 14 days after the onset of injection. By days 16 and 18, there was almost complete regression of tumors in the imatinib and SkE-treated mice. Finally, histological slides of tumors clearly showed dephosphorylation of ERK in tumors collected from SkE-treated mice at day 18 (Figure 5C). Clearly, there was also a visible decrease in the number of K562 cells present in the tumors of SkE-treated animals. Taken collectively, these data demonstrate that SkE (1 mg/kg) is as effective as imatinib (60 mg/kg), the leading compound for treating CML patients, which is used to inhibit CML cell growth in vivo. Moreover, the effect of SkE in vivo relied on ERK1/2 dephosphorylation.

## DISCUSSION

The Ras/Raf/MEK/ERK cascade is a very attractive target in cancer therapy. Indeed, numerous solid and hematopoietic tumors exhibit activation of this pathway following genetic alterations either in upstream signaling molecules, such as receptor tyrosine kinases and oncogenic fusion proteins, or in overexpression of one of the elements of this pathway [2, 3]. The upstream regulator of the cascade, Ras, is mutated in 20 to 30% of human cancers. Of note, the frequency of K-Ras mutations is very high in advanced pancreatic cancers [23]. Mutations in the downstream kinase B-Raf are also frequently found in cancers. This is well exemplified in melanoma, in which B-Raf mutations are present in 50-70% of patients, and in HCL, in which the frequency of B-Raf mutations is close to 100%. In both cases, the B-Raf-V600E mutation is consistently detected. The downstream kinase MEK1/2 is mutated at a low frequency in some cancers, and, to date, there is no evidence of mutation in the downstream kinase ERK1/2. In addition to mutations in various elements of the cascade, the Ras/Raf/MEK/ERK pathway is found to be activated in a very large number of hematopoietic and solid tumors [2, 24, 25]. For instance, constitutive activation of the pathway is also observed independent of activating mutations in solid and hematopoietic malignancies, including CML, in which the BCR-ABL fusion oncoprotein (which drives the pathology) is responsible for ERK1/2 activation. Importantly, our group and others have also reported a high constitutive activation of ERK1/2 in several tyrosine kinase inhibitor-resistant CML cell lines [26-28]. Of note, SkE was also found to be highly effective at killing these tyrosine kinase inhibitor-resistant CML cell lines (data not shown).

The present study was conducted to determine whether SkE could be used as an antitumoral agent. We show here that very low doses of SkE efficiently inhibited the growth of several solid and hematopoietic cancer cell lines, including melanoma and CML cell lines. Of note, SkE was highly efficient in cells exhibiting constitutive activation of the Ras/Raf/MEK/ERK pathway. For instance, SkE was found to be ten-fold more effective in melanoma cell lines carrying the V600E B-Raf mutation than in melanoma cell lines that did not carry a mutation (Supplementary Figure 1). In addition, SkE was also very potent in CML cell lines exhibiting constitutive activation of the MAP kinase pathway following the expression of the BCR ABL fusion protein.

To decipher the mechanism of action of SkE, we used both melanoma and CML cell lines and primary cells from patients diagnosed with melanoma and HCL and demonstrated that low concentrations of SkE interfere with the activation of ERK, as shown by the clear inhibition of ERK1/2 phosphorylation. We next sought to identify the specific point in the Ras/Raf/MEK/ERK pathway affected by SkE in different cellular models with either constitutive or inducible MAPK kinase activation. Importantly, SkE impaired the activities of both MEK and B-Raf. By contrast, SkE failed to affect Ras activity, strongly suggesting that the drug acts at the level of B-Raf to inhibit the ERK pathway. At present, it is not known whether SkE also affects the activity of other Raf isoforms, including A-Raf and C-Raf.

Importantly, chloroquine, an anti-malarial drug with a chemical structure different from SkE, has previously been reported to inhibit ERK activation [29]. However, the chloroquine dose necessary to achieve complete inhibition of ERK in human peripheral blood monocytes in this study was 1000 times higher than the one used in the present study for SkE in melanoma and CML cell lines. This very high potency of SkE in inhibiting B-Raf prompted us to assess its activity in melanoma cell lines carrying B-Raf-V600E mutations and in primary cells from HCL patients who consistently carried this mutation. SkE potently inhibited the growth and clonogenic potential of both cell lines, confirming the very potent anti-tumoral effect of this drug, particularly in cells exhibiting constitutive activation of the Ras/Raf/MEK/ERK cascade.

Owing to its ability to inhibit lysosomal protease, chloroquine is often used as an inhibitor of autophagy, a catabolic process that can favor cell survival in adverse conditions, such as cellular stress and nutrient deprivation [30]. In this line, the inhibition of autophagy can sensitize cancer cell lines to chemotherapy, and several clinical trials have been initiated that include chloroquine as a second-line therapeutic agent in different forms of cancers [31, 32]. However, the findings presented herein clearly establish that an optimal concentration of SkE failed to affect the lipidation of LC3, arguing against an effect of SkE on autophagy induction when used as a single drug.

In the present study, we also demonstrated that SkE drastically reduced the growth of CML cells in athymic mice. A dose as low as 1 mg/kg of SkE was sufficient to inhibit the growth of K562 cells, whereas 60 mg/kg of imatinib mesylate, the leading treatment for CML, was required to obtain a similar effect. These results clearly show that SkE has an excellent in vivo bioavailability in mice. Moreover, our results strongly suggest that the antiproliferative and proapoptotic effects of SkE are intimately linked to its ability to interfere with the MAP kinase cascade. This was confirmed by our analysis of tumor histological slides from athymic mice grafted with K562 CML cell lines, which clearly showed a complete inhibition of ERK1/2 phosphorylation in SkE-treated mice.

Finally, we also present evidence that SkE is highly effective at circumventing dabrafenib (GSK2118436) resistance in melanoma cell lines. Dabrafenib is a potent B-Raf inhibitor currently used in phase III studies for metastatic melanoma. It has been reported that dabrafenib initially induced complete remission in patients with metastatic melanoma [14]. However, following this initial beneficial response, all of the patients relapsed. Relapses are likely due to the reactivation of the MAPK pathway and, accordingly, MEK inhibitors such as U0126 can efficiently resensitize dabrafenib-resistant cell lines in vitro. Our group and others have recently reported that the B-Raf inhibitor vemurafenib (PLX-4720) is very effective in HCL patients who carry the B-Raf V600E mutation, inducing complete remission and the restoration of normal blood cell counts and hemoglobin concentration in patients with refractory HCL [11]. Another important finding of the present study is that low concentrations of SkE can inhibit the growth of primary cells from HCL patients more efficiently than vemurafenib (i.e., inhibition occurs in the high nanomolar range versus the micromolar range).

In conclusion, we describe here for the first time the unusual ability of the new compound SkE to inhibit B-Raf activation not only in melanoma and HCL but also in CML cell lines exhibiting constitutive activation of the ERK pathway. In addition, we show that this drug is highly effective at inhibiting HCL-patient-derived primary blood cells carrying this mutation and at inhibiting melanoma melanoma cell line with acquired resistance to the B-Raf inhibitors PLX-4720 and GSK2118436. Finally, we also show evidence that SkE at very low doses is highly effective in a preclinical murine model of CML. Collectively, our findings show that SkE could be a new weapon in the armamentarium of drugs targeting cancers that exhibit constitutive activation of the ERK pathway and that SkE warrants testing in humans.

## MATERIALS AND METHODS

### Reagents and antibodies

RPMI 1640 and DMEM media as well as fetal calf serum (FCS) were purchased from Lonza (Walkersville, MD, USA). Sodium fluoride, orthovanadate, phenylmethylsulfonyl fluoride, aprotinin and leupeptin were purchased from Sigma (Saint-Louis, MO, USA). Imatinib was purchased from Enzo Life Sciences (Villeurbanne, France). U0126 was purchased from Tocris (Bristol, UK). PLX-4720 was purchased from *Selleck Chemicals* (Houston, TX, *USA*). Anti-C-Abl, anti-MEK1/2, anti-Hsp90 and anti-Hsp60 antibodies were purchased from Santa Cruz Biotechnology (Tebu-Bio, Le Perray en Yvelines, France). Anti-phospho-Abl (Tyr245), anti-phospho-STAT5, anti-phospho-Crkl (Tyr207), anti-PARP, anti-phospho-S6 Ribosomal Protein (Ser235/236), anti-S6 Ribosomal Protein, anti-phospho-ERK1/2, anti-ERK1/2, anti-phospho-MEK1/2, anti- phospho-B-Raf, anti-B-Raf and anti-LC3b were purchased from Cell Signaling Technology (Danvers, MA, USA). HRP conjugated anti-mouse, anti-rabbit and anti-goat antibodies were purchased from Dakopatts (Glostrup, Denmark).

### Cell lines

The human CML K562 cell line was provided by ATCC and was grown at 37°C under 10% CO2 in RPMI 1640 medium supplemented with 5% FCS (Gibco BRL, Paisley, UK) and 50 units/ml of penicillin, 50 μg/ml streptomycin and 1 mM sodium pyruvate. 293 RAF/ER cells are a derivative of HEK-293 (ATCC: CRL1573) cells that stably express a fusion protein comprising the catalytic domain of Raf-1 and the hormone-binding domain of the estrogen receptor. 293 RAF/ER cells were cultured in DMEM without phenol red, supplemented with 10% heat inactivated FCS, as described previously [17]. The 451Lu melanoma cells, which are sensitive or resistant to PLX-4720, were grown in DMEM supplemented with 10% FCS.

### Measurement of cell metabolism (XTT)

Cells (15×10^3^ cells/100 μl) were incubated with the different effectors for the times indicated. A total of 50 μl of XTT reagent (sodium 3′-[1-(phenylaminocarbonyl)-3,4-tetrazolium]-bis(4-methoxy-6-nitro)benzene sulfonic acid hydrate) was added to each well. Absorbance of the formazan dye produced by metabolically active cells was measured at 490 nm as described previously [18]. Each assay was performed in quadruplicate.

### Western Blot

Western blot analyses have been previously described in detail [19].

### Phosphoprotein array analysis

*K562 cells were treated* (or not treated) with 250 nM SkE for *2 hours*. Cells were rinsed with cold PBS and lysed as described for Western blot analysis. Cell lysates were clarified by centrifugation (10,000 g for 5 minutes at 4°C), and the protein levels were normalized using the Bradford assay. Then, 150 μg of cell extracts was left on the chip as described in the RTK Pathscan array kit from Cell Signaling Technology (Danvers, MA, USA). After incubation and successive steps of washing, the arrays were dried and imaged using a Fujifilm LAS-3000 Imaging System. Duplicate spot intensities were quantified from each array image using the Image J quantification software (U.S. National Institutes of Health, Bethesda, MD, USA).

### Primary cell isolation

Blood samples were collected from patients newly diagnosed with CML or HCL as part of an institutionally approved cellular sample collection protocol. Informed consent was obtained according to institutional guidelines. Mononuclear cells were isolated from blood samples by density centrifugation (Ficoll-Paque™ Plus), washed with PBS, 5% SVF, and 2 mM EDTA, and then resuspended in cell culture medium (IMDM, 10% fetal bovine serum) and incubated overnight at 37°C in a 5% CO2 incubator. CML cells were labeled with CD34 microbeads isolated by magnetic positive selection (StemSep™ Human CD34 Selection Kit; StemCell, Vancouver, BC, Canada). Purity was estimated to be at least 90% by FACS analysis. Experiments were performed using an IMDM (10% fetal bovine serum) for CML and HCL cells.

### Colony formation assay

SkE was added to the K562 CML cell lines (10^3^ cells/ml) growing in semi-solid methylcellulose medium. MethoCult H4100 was used for cell lines (StemCell Technologies Inc., Vancouver, Canada). Colonies were detected after 10 days of culture by adding 1 mg/ml of 3-(4,5-dimethylthiazol-2-yl)-2,5-diphenyltetrazoliumbromide (MTT) reagent and were scored by Image J quantification software (U.S. National Institutes of Health, Bethesda, MD, USA).

### Microscopic analysis of tumors

Tumors from control or SkE-treated mice were removed, frozen and cut in preparation for immunostaining. Slides containing a representative section of each tumor were fixed, permeabilized and incubated with anti-phospho-ERK 1/2 or anti-ERK antibodies diluted in PBS and 1% BSA at RT for 1 h. Then, cells were washed and incubated with a secondary anti-Rabbit antibody. Finally, DAPI was used to label the nuclei, and the slides were mounted and then analyzed under a fluorescence microscope (LEICA TCR5500, Leica, Somls, Germany).

### Tumor regression experiments in athymic Mice

Female athymic NMRI Mice (Janvier, Le Genest Saint Ile, France) were randomized into 3 experimental groups, each containing 7 animals. Two sets of animals received a 200 μl injection of 5.10^6^ K562-Luc leukemia cells in both flanks. When tumors reached 100 mm^3^, the animals were injected intraperitoneally with vehicle (PBS), Imatinib or SkE at dose levels of 60 mg/kg and 1 mg/kg body weight, respectively. The growth of the leukemic cells comprising the tumor was visualized in the animal at different days after intraperitoneal injection of 30 mg/kg luciferin (Caliper Life Sciences) by bioluminescence imaging with a Photon Imager (Biospace Lab), as described elsewhere [20]. The in vivo study was conducted according to French legislation on laboratory animal use and care (N°2001-464).

### Measurement of cell death

Following U0126, PLX-4720 or SkE treatment, HCL cells were stained with propidium iodide, and the stained cells were analyzed with a cytometer.

### Extraction and purification of simalikalactone E

SkE was extracted and purified from Quassia amara as previously described [1].

### Pull-down of the activated form of Rho GTPase

K562 cells were incubated at 37°C with 250 nM SkE for the indicated times. Ras activity was determined after GST-pull-down. Ras-GTP levels were determined using GST-c-Raf RBD to pull down active GTP-bound Ras from cell extracts by glutathione beads. The beads were washed 4 times and subjected to SDS/PAGE (12% polyacrylamide). Ras and Phospho-ERK1/2 proteins were detected by Western blot analysis as described previously [21].

### Statistical analysis

All data are presented as the mean ± SD of at least three independent determinations. P-values were determined using the Prism V5.0b software (GraphPad, La Jolla, CA, USA). Unless stated otherwise in the figure legend, comparisons of the different groups were made with the one-way ANOVA test with Bonferroni correction. P-values of 0.05 (*), 0.01 (**) and 0.001 (***) were considered statistically significant.

## Supplementary Figures


