# Supplementary material for: Simalikalactone E (SkE), a new weapon in the armamentarium of drugs targeting cancers that exhibit constitutive activation of the ERK pathway
Source: Oncotarget. 2012 Dec 30;3(12):1688–99. doi: 10.18632/oncotarget.791 (PMC3681504; doi:10.18632/oncotarget.791)
Supplement: Supplementary file 1 [file oncotarget-03-1688-s001.pdf]

Simalikalactone E (SkE), a new weapon in the armamentarium of drugs targeting cancers that exhibit constitutive activation of the ERK pathway – Robert et al

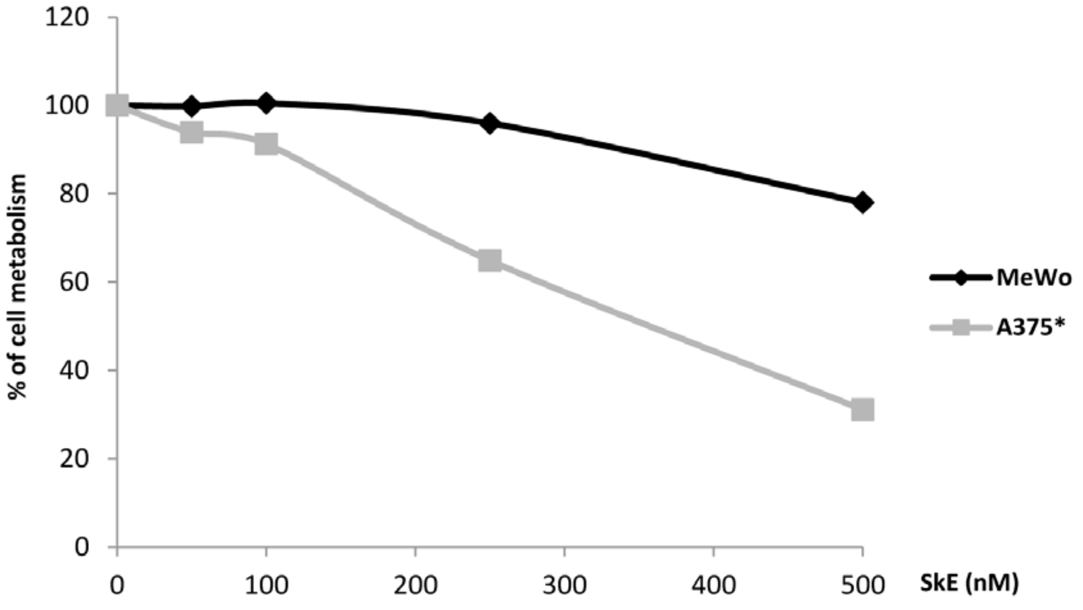

Supplementary Figure 1
